# Gout With Associated Cutaneous AA Amyloidosis: A Case Report and Review of the Literature

**DOI:** 10.3389/bjbs.2023.11442

**Published:** 2023-06-13

**Authors:** G. E. Orchard

**Affiliations:** Synnovis Analytics, St. John’s Histopathology Department, St. Thomas’ Hospital, London, United Kingdom

**Keywords:** gout, AA (secondary) amyloidosis, urate crystals, amyloid, chronic inflammation

## Abstract

Gout with associated AA amyloidosis is an unusual finding. This form of amyloid is associated with chronic inflammatory changes often associated with amyloid deposits in the urine, as well as tissue involvement, and organ enlargement in some cases. The large majority of cases in the literature to date refer to gout with AA amyloid within the kidney. However, this is not exclusive, with reports in the liver, gastrointestinal tract, adrenal glands rectum, skin, and subcutaneous fat. The pathophysiological association between these two disease processes is open to debate. The employment of specific anti-inflammatory treatments is believed to have an impact on reducing the incidence of AA amyloidosis in some gout cases–notably the use of colchicine in cases of clinically defined gout attacks. However, this is by no means a universal finding. Here we report on a cutaneous case of gout with AA amyloidosis in a 73-year-old man Included in this case study is a review of the other 16 cases reported within the literature in an attempt to clarify the associated pathophysiological process between these two diseases and the anti-inflammatory treatment regimens employed which may impact the occurrence of AA amyloidosis.

## Introduction

Gout is a form of inflammatory arthritis. It commonly affects one joint at a time, often digits, especially the big toe. There is considerable pain and discomfort along with swelling, redness, and tenderness of the surrounding affected tissue. The condition is caused when urate (monosodium) crystals accumulate within the affected joint which subsequently causes inflammation and intense pain [1–4]. The urate crystals accumulate due to a build-up of uric acid in the blood as a result of the breakdown of purines which occur naturally. Uric acid is normally discharged through the kidneys into the urine. However, a build-up of uric acid may occur due to a number of different factors such as dietary issues, i.e., consuming a diet rich in meat and shellfish and drinking sweetened drinks or alcoholic beverages in excess [3, 4]. Underlying medical conditions such as high blood pressure, diabetes, and heart and kidney diseases are also risk factors as are obesity, family history, and predisposition to gout [1–5]. Gout may also occur following recent surgery or trauma [6] and is generally more prevalent in men than women [1, 2].

Amyloidosis occurs when the normal alpha-helical structure of proteins loses its three-dimensional structure, and, as a result, becomes twisted and clumps together to form a beta-pleated sheet conformation of otherwise termed amyloid fibrils. These accumulate within tissues and organs and effectively cause disruption to normal physiological and biochemical activities within the body [7–9]. AA (secondary or serum A) amyloidosis is a condition that arises when there are high levels of inflammation within the body. As such it is regarded as a serious complication of any chronic inflammatory conditions such as rheumatoid arthritis, Crohn’s disease, or ulcerative colitis [10]. The association of AA amyloidosis with gouty arthritis is however unusual and rare [11–15]. Here we report a case of a 73-year-old man with renal disease, with gout and cutaneous AA amyloidosis from a tophus removed from the right elbow.

## Case Study

**FIGURE 1:**
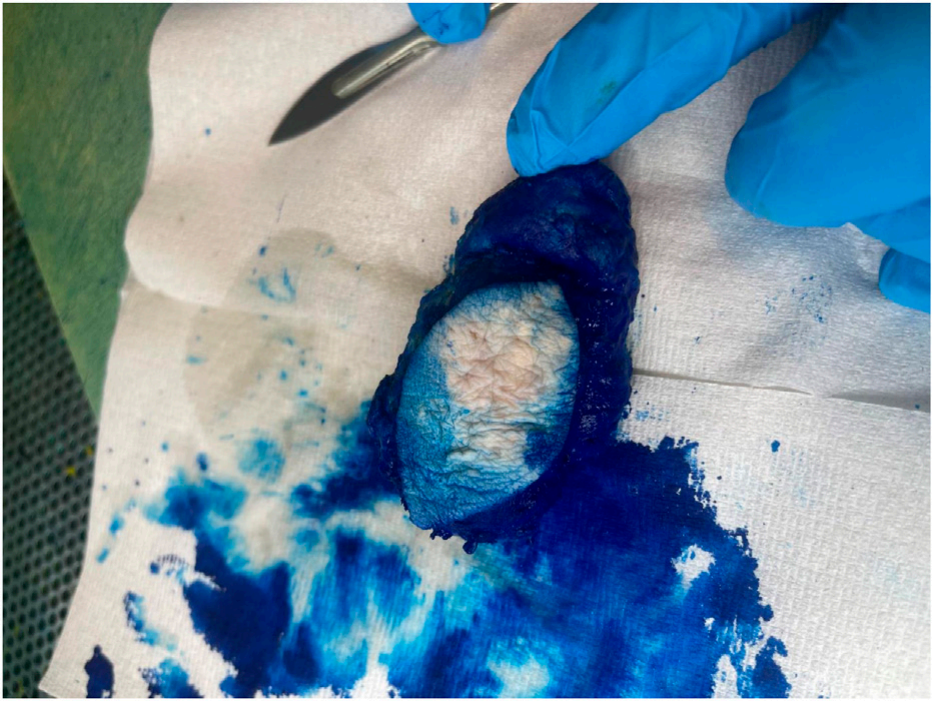
Macroscopic appearance of the gout tophus removed from the right elbow of a 73-year-old man.

**FIGURE 2:**
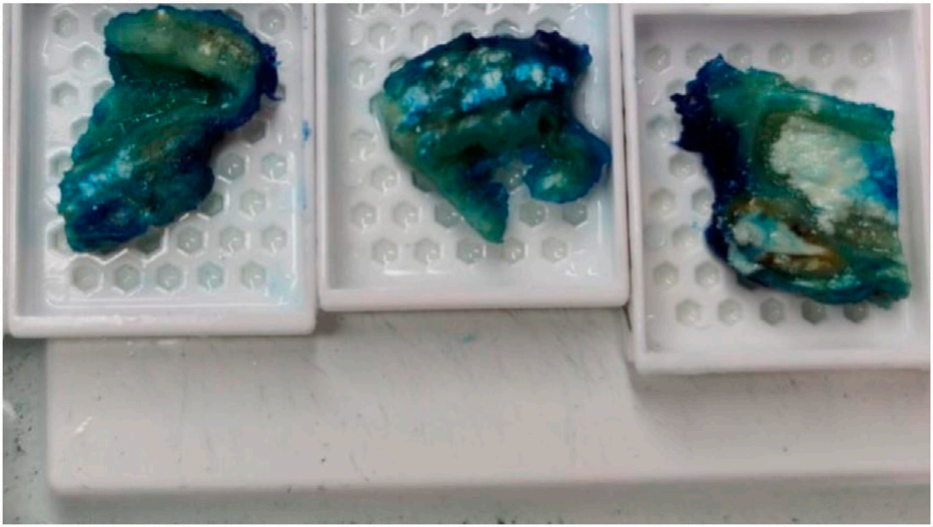
On grossing the sample amorphous deposits of white material were seen predominantly within the reticular dermal area and extended down to the subcutaneous fat.

**FIGURE 3:**
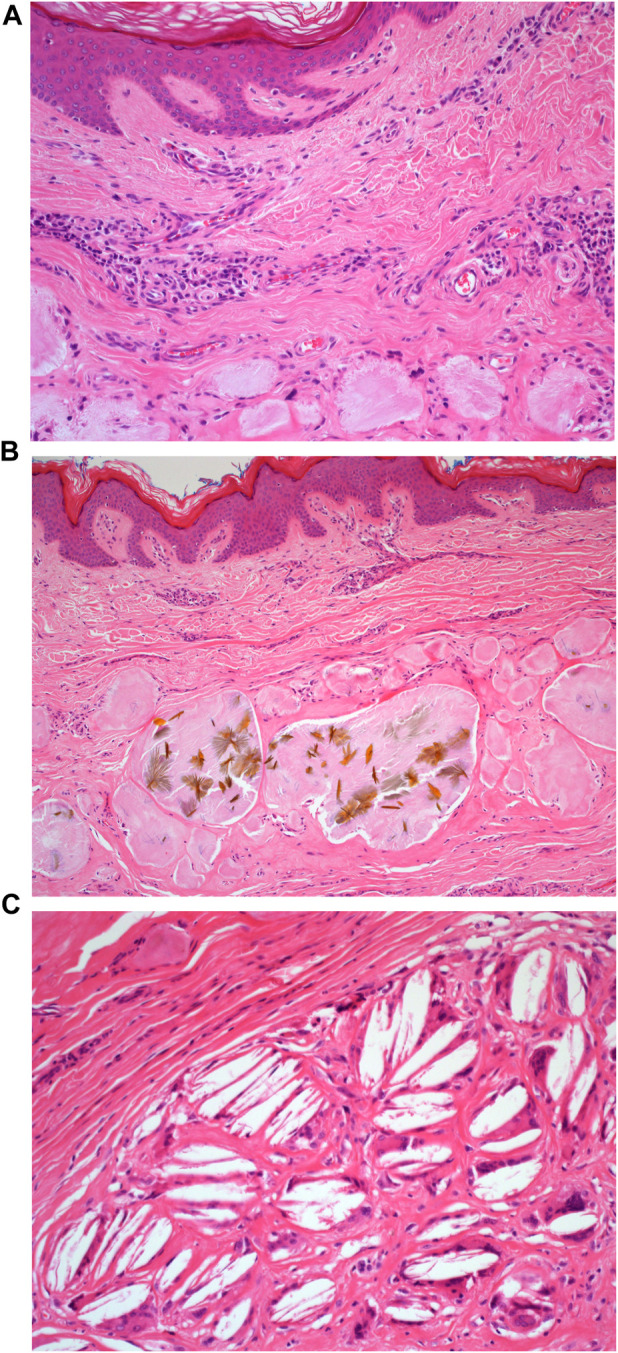
**(A)**: H&E staining of the microscopic appearance of the dermal amorphous eosinophilic deposits. Mag ×20. **(B)**: H&E staining of the microscopic appearance of the crystalloid structures. Mag ×20. **(C)**: H&E staining demonstrating the appearance of cholesterol clefts. Mag ×40.

**FIGURE 4:**
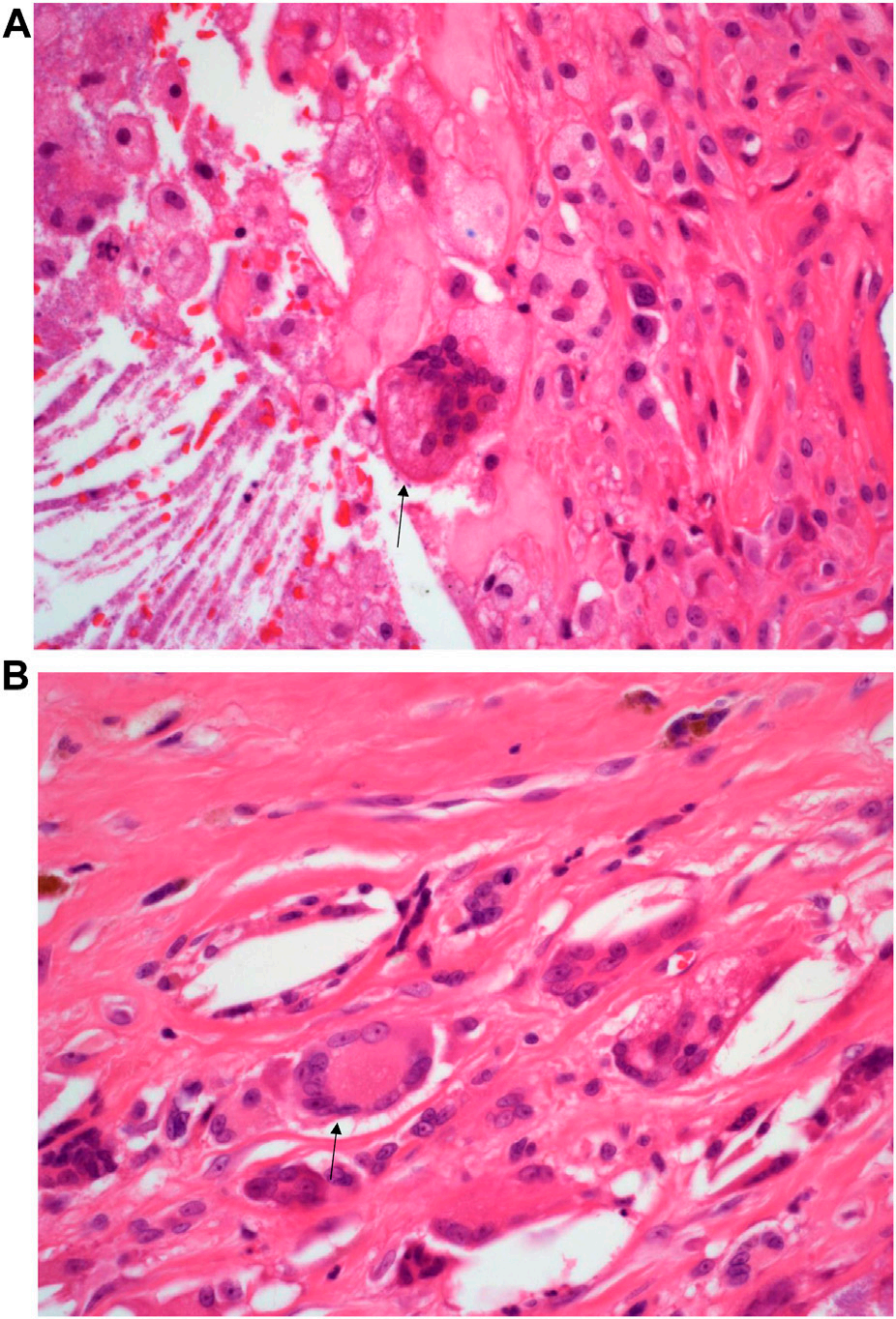
**(A)**: H&E staining of the inflammatory infiltrate composed of foreign body multi-nucleated giant cells (black arrow). Mag ×40. **(B)**: H&E staining of the inflammatory infiltrate composed of Touton type multi-nucleated giant cells (black arrow) Mag ×40.

**FIGURE 5:**
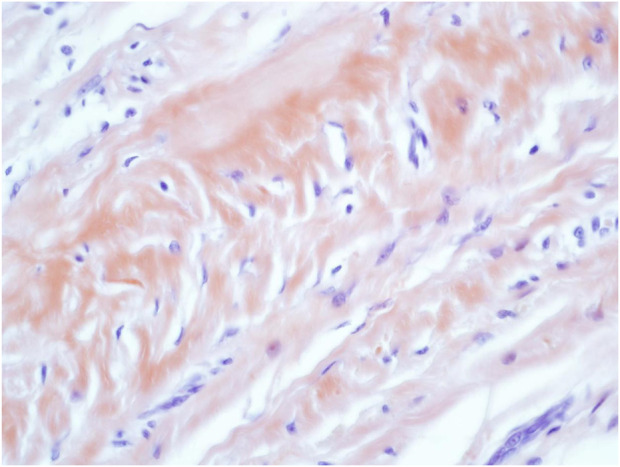
Congo Red (CR) staining of the amorphous AA amyloid deposits within the reticular dermal area Mag ×40.

**FIGURE 6:**
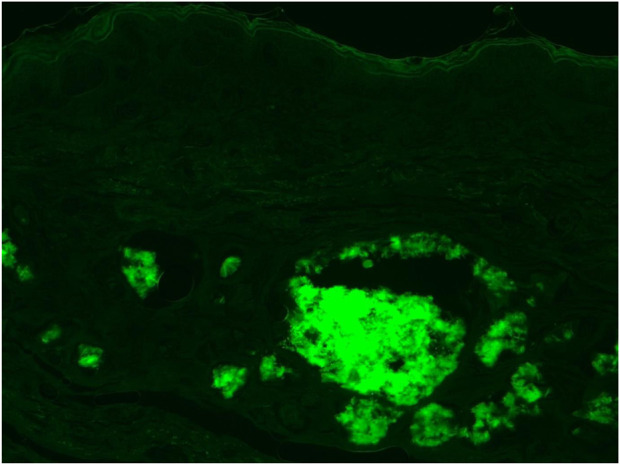
Fluorescent microscopy of Thioflavin-T (Thio-T) staining of the AA amyloid deposits found within the dermal area. Mag ×40.

**FIGURE 7:**
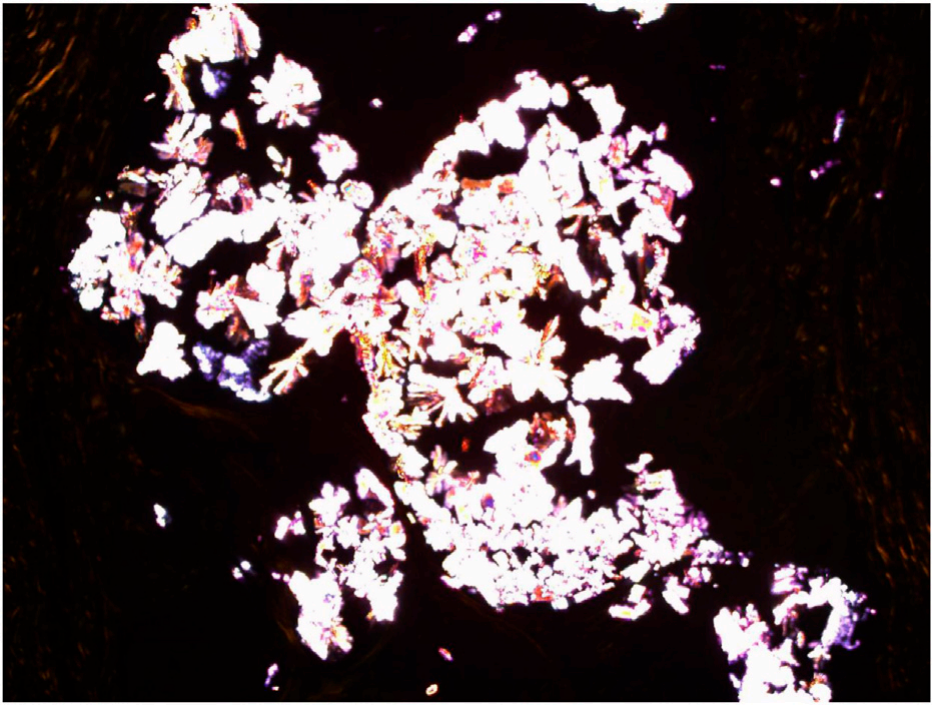
Congo Red (CR) staining with cross polarisers demonstrating the Uric acid crystalloid structures. Mag ×30.

A 73-year-old man with impaired renal function and a previous history of gout presented with a gout tophus on his right elbow. Physical examination showed extensive inflammation of the right elbow area. The laboratory findings included an abnormal renal profile with creatinine level 179 (reference 64–104 µmol/L), sodium level 140 (reference 133–146 mmol/L) which is within the normal range, and potassium level 5.4 (reference 3.5–5.3 mmol/L). The urea level was 11.6 (reference 2.5–7.8 mmol/L) and the Erythrocyte sedimentation rate was 100 mm/h. Histopathological examination of the removed gout tophus from the right elbow demonstrated a fibrofatty mass measuring 70 × 46 × 22 mm (Figure 1). Macroscopically on slicing the tophus, areas of amorphous crystalloid material were seen and identified predominantly within the reticular dermal area (Figure 2). Macroscopically, sections revealed an epidermis with hyperkeratosis and regular acanthosis. Within the reticular and subcutaneous tissue there were areas of amorphous pale eosinophilic material with associated crystalloid structures (Figures 3A, B) displaying a feathery appearance with characteristic needle-shaped spaces (Cholesterol clefts) (Figure 3C). There were also areas of calcification seen. Of note was the presence of granulomatous inflammation with the presence of histiocytes, giant cells (foreign body and Touton types) (Figures 4A, B arrowed), plus the presence of fibrosis. The amorphous material extended to the papillary dermis and special stains for amyloid were conducted which included Congo Red (CR) (Figure 5) and Thioflavine–T (Thio-T) (Figure 6). Using cross-polarized light the crystalloid structures (Figure 7) within the amorphous material located within the papillary and reticular dermal areas exhibited birefringence in the CR-stained sections and was positive with Thio T confirming the presence of amyloid.

## Discussion

Gout is a fairly common disease and an ancient one, being described by the Egyptians in 2640 BC as lesions compromising the first metacarpophalangeal joints [16]. To give an idea of how common the occurrence of gout is–an estimated 6.1 million adults within the United States (approximately 2.5%) of the adult population are diagnosed with gout [16]. However, gout with associated amyloidosis is rare in comparison [10] (Table 1). AA amyloidosis causes complications in chronic infections and inflammatory diseases like rheumatoid arthritis. Since the pathophysiological connection between gout and amyloidosis is not understood there have been variable approaches to treatment regimens [10, 15]. Some of these could control either the gout or the amyloidosis, but there is no evidence that describes the successful treatment of both conditions [15]. The large majority of the cases reported in the literature had tophaceous gout for at least a period of 10 years [10–14] and were not using colchicine (Colcrys, Gloperba, Mitigare) on a regular basis. Colchicine is an anti-inflammatory drug that reduces the effects of gout and is often preferred over nonsteroidal anti-inflammatory drugs ibuprofen or naproxen or more powerful options such as indomethacin or celecoxib, or corticosteroids such as prednisone.

**TABLE 1:** Key features of patients with gout and AA amyloidosis from the literature including our case (patient 16).

Case number and references	Age	Sex	Type of gout and duration	Site of amyloid deposition	Family history	Treatment and associated possible causes of amyloid
Levo et al. [8]	46	Male	Chronic Tophaceous gout 32 years	Kidneys, adrenals	Yes	Colchicine
Levo et al. [8]	39	Male	Monoarthritis gout 1 year	Kidneys	No	Colchicine
Lens et al. [9]	49	Male	Chronic Tophaceous gout 20 years	Kidneys, liver, and subcutaneous fat	No	No Colchicine
Guma et al. [12]	46	Male	Chronic Tophaceous gout 20 years	Rectum and kidneys	No	No Colchicine
Vernerova et al. [14]	56	Male	Chronic Tophaceous gout 15 years	Kidneys	Yes	Allopurinol
Vernerova et al. [14]	44	Male	Chronic Tophaceous gout 10 years	Rectum, Kidneys	Yes	Allopurinol and colchicine (after diagnosis of AA amyloidosis)
Rubinow and Sonnemblick [11]	85	Male	Chronic Tophaceous gout 10 years	Rectum and kidneys	No	Colchicine (after diagnosis AA amyloidosis)
Talbott and Terplan [7]	42	Male	Chronic Tophaceous gout 25 years	Widespread	No	Azathioprine (Pyelonephritis)
Talbott and Terplan [7]	23	Male	Chronic Tophaceous gout 9 years	Kidneys	Yes	No Colchicine Probenecid dialysis (tuberculosis)
Talbott and Terplan [7]	32	Male	Monoarthritis 4 years	Kidneys	No	Tuberculosis meningitis No colchicine
Talbott and Terplan [7]	72	Male	Chronic Tophaceous gout 37 years	Kidneys	No	High-dose salicylates and on allopurinol dialysis
Gaviria et al. [16]	47	Female	Monoarthritis gout 1 year	Kidneys and liver	No	Allopurinol and Colchicine
Gromova and Tsurko [15]	62	Male	Chronic Tophaceous gout 36 years	Kidneys GI tract, adrenal glands	No	Colchicine, prednisone, allopurinol
Jacobelli et al. [13]	61	Male	Chronic Tophaceous gout	Kidneys	No	Unknown
Ter-Borget al. [10]	57	Male	Chronic Tophaceous gout	Kidneys	No	Colchicine after gout attack
*Last case*	73	Male	Chronic Tophaceous gout 10 years	Kidneys and skin	No	Colchicine after gout attack

Amyloid deposits are due to an elevation in acute-phase reactants or proteins, which often occur in and around gout tophi. However, not all of the reactant proteins share the amyloidogenic profile thus in part explaining why so few cases are perhaps reported [15]. AA amyloid results from the extracellular deposition of the sub–acute-phase reactant serum amyloid A (SAA) protein as insoluble amyloid fibrils. When this process is prolonged the liver will produce high levels of SAA resulting in the trigger that drives the development of AA amyloidosis [15]. It is also possible that the inflammatory mediators that trigger and accentuate the inflammatory response may differ between gout and associated amyloid conditions like rheumatoid arthritis. Rheumatoid arthritis is a long-term chronic inflammatory condition whereas gout, although also a chronic inflammatory condition is generally a more active and florid inflammatory response in keeping with the clinical spectrum of progressive inflammation which suggests perhaps the difference between the respective inflammatory pathways could also account for the absence of amyloid deposits within the majority of gout cases [1, 10].

Finally, since the routine treatment for patients with gout is the use of colchicine and many of the cases reported within the literature for clinically defined reasons did not have colchicine administered, it is thus that one could speculate that the lack of administration of colchicine is a contributing factor in the development of amyloid in this case as in others reported [10, 17]. It is known that colchicine does prevent casein-induced amyloidosis in mice [18]. However, conversely, it is also the case that the majority of patients with gout are only administered colchicine during a gout attack and not as an ongoing regular treatment option. Cumulatively this may suggest that the low incidence of amyloidosis in gout may not be directly associated with the administration of this drug, but may be related to a number of clinical and pathological multifactorial issues. Further studies are therefore required to understand the pathophysiological relationship between gout with AA amyloidosis.

Summarising the data in the literature along with the evidence from this case study, there are only 16 reported cases of amyloidosis associated with gout. The incidence is rare. This case is also unusual as most cases of chronic gout with amyloidosis occur in the kidney or rectum. Investigations of kidney AA in this case are under review. It is unusual to find cases of gout with AA amyloidosis within the skin [16].

## Summary Table

• The association of gout with AA amyloidosis is rare. There are currently only 15 reported cases within the literature. In most cases, the AA amyloid is found within the kidneys. The presence of AA amyloid in these cases appears to be associated with the anti-inflammatory drug treatment given to these patients, most notably during a gout attack. However, the physiological and clinical-pathological correlation between gout and AA amyloidosis is not fully understood.
• Here we report an unusual case of gout with AA amyloidosis occurring in a 73-year-old man and affecting the skin, in which the amyloid presented whilst the patient had been previously treated with the anti-inflammatory drug colchicine. The association between these two disease processes needs further elucidation, as most publications to date are of isolated case reports.
• This work represents an advance in biomedical science because it demonstrates that the association between these two disease processes affects multi-organ sites and that the simultaneous occurrence in the skin is unusual.

## Data Availability

The original contributions presented in the study are included in the article/supplementary material, further inquiries can be directed to the corresponding author.
